# Electronic resonances in broadband stimulated Raman spectroscopy

**DOI:** 10.1038/srep18445

**Published:** 2016-01-05

**Authors:** G. Batignani, E. Pontecorvo, G. Giovannetti, C. Ferrante, G. Fumero, T. Scopigno

**Affiliations:** 1 Universitá di Roma “La Sapienza”, Dipartimento di Fisica, Roma, I-00185, Italy; 2 Universitá degli Studi dell’Aquila, Dipartimento di Scienze Fisiche e Chimiche, L’Aquila, I-67100, Italy; 3 Istituto Italiano di Tecnologia, Center for Life Nano Science @Sapienza, Roma, I-00161, Italy

## Abstract

Spontaneous Raman spectroscopy is a formidable tool to probe molecular vibrations. Under electronic resonance conditions, the cross section can be selectively enhanced enabling structural sensitivity to specific chromophores and reaction centers. The addition of an ultrashort, broadband femtosecond pulse to the excitation field allows for coherent stimulation of diverse molecular vibrations. Within such a scheme, vibrational spectra are engraved onto a highly directional field, and can be heterodyne detected overwhelming fluorescence and other incoherent signals. At variance with spontaneous resonance Raman, however, interpreting the spectral information is not straightforward, due to the manifold of field interactions concurring to the third order nonlinear response. Taking as an example vibrational spectra of heme proteins excited in the Soret band, we introduce a general approach to extract the stimulated Raman excitation profiles from complex spectral lineshapes. Specifically, by a quantum treatment of the matter through density matrix description of the third order nonlinear polarization, we identify the contributions which generate the Raman bands, by taking into account for the cross section of each process.

Spontaneous Raman (SR) spectroscopy is a powerful technique which has been widely exploited to investigate complex molecular and solid state systems. Its inherent sensitivity to the vibrational properties, allows to extract structural and dynamical information, despite a relatively weak cross section. An enhancement of the Raman signal, essential for studies at low concentrations or in low cross section compounds, is achieved by Resonance Raman Spectroscopy (RRS), in which the Raman excitation wavelength is tuned to match the energy of an electronic transition of the system^1^,^2^. By varying the resonance condition, RRS enables to selectively isolate the contributions from different chromophores, providing detailed information with structural resolution. Even under resonance conditions, however, the SR cross section is several orders of magnitude smaller than the fluorescence emission, which can easily overwhelm Raman signals. Furthermore, SR is an incoherent process and the generated radiation is spread over a 4*π* steradian solid angle. One of the most critical limitations of SR arises when it is used as a probe technique for time resolved studies of ultrafast structural dynamics. In this case, the temporal and energy resolution is fundamentally constrained by the Fourier transform limit, i.e. by the time-bandwidth product of the light pulse, which dictates 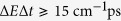
3,4,5 thus preventing the access to sub-picosecond dynamics with adequate spectral resolution.

Recently, the development of novel nonlinear vibrational spectroscopies has allowed to mitigate such limitations. In particular, broadband Stimulated Raman Spectroscopy (SRS) provides high intensity, fluorescence background free coherent signal. In SRS the sample is interrogated by a pair of overlapped narrowband picosecond Raman Pulse (RP) and broadband femtosecond Probe Pulse (PP). Vibrational spectra are generated on top of the highly directional PP, hence the incoherent fluorescence background is efficiently suppressed. Adding an ultrashort photo-excitation pulse, which triggers a photochemical process, turns SRS into Femtosecond Stimulated Raman spectroscopy (FSRS)^6^,^7^,^8^,^9^,^10^,^11^,^12^,^13^,^14^,^15^, the ideal tool to study structural changes in ultrafast photophysical and photochemical processes, providing both femtosecond time resolution and high spectral resolution^16^,^17^. The Raman bands in SRS spectra are induced by the third order Raman susceptibility and arise as a modification of the PP spectral profile. Therefore, it is convenient to define the stimulated Raman gain as: 
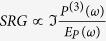
18,19, where *E*_*P*_(*ω*) is the spectral envelope of the PP, *P*^(3)^ (*ω*) is the third order polarization induced in the sample and 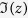 indicates the imaginary part of *z*. As in Spontaneous Raman, the SRS features arise both to the red and to the blue side of the Raman pulse. While the red component of the SRS spectrum is always a positive signal and corresponds to a Stokes process in Spontaneous Raman, the blue component has no spontaneous (Antistokes) counterpart^20^ and shows a rich lineshape dependence on the resonance condition induced by the RP wavelength. This latter peculiar behavior has only recently been addressed in a few works^21^,^22^,^23^,^24^ and an overall experimental and theoretical analysis has not yet been proposed, clarifying the exact mechanism which rules the link between vibrational lineshapes and resonance condition, as well as the enhancement of the cross section in the blue side respect to the red side.

In this paper, taking advantage of a simple diagrammatic approach to calculate the third order nonlinear polarization induced in the system, we identify the physical processes ruling the red and blue signals of SRS spectra. Hence, the behavior of broadband SRS spectra and their dependence on resonance conditions are fully understood and a general way to extrapolate the Raman Excitation profile for the blue side of the spectrum is proposed. Our approach is experimentally validated by reproducing resonance effects on a paradigmatic heme protein, namely ferrous Myoglobin (Mb), probed by SRS across the Soret absorption band.

## Result and Discussion

Broadband Stimulated Raman spectroscopy exploits the macroscopic nonlinear third order polarization (*P*^(3)^) induced in a Raman-active medium by the spatial and temporal overlap of a narrowband RP and femtosecond PP pulses, to obtain high quality Raman spectra. The nonlinear process perturbs the Raman and Probe fields and produces a transfer of photons between them, which results in the generation of Raman signals on top of the PP. In off resonance condition the red side of the Stimulated Raman spectrum (relative to the RP) is analogous to a SR Stokes spectrum, i.e. it consists of positive peaks (gains). The blue side shows negative peaks (losses) and, at variance with SR, their intensity relative to the red side does not retain any information on temperature, as recently pointed out in ref. 20. Under resonance condition, the SRS response features a more complex behavior. The spectral profile of the red side Raman bands does not significantly change, albeit the intensity undergoes a resonance enhancement with a wavelength dependence very similar to SR excitation profile. Remarkably, the lineshapes of the blue side bands are totally different, evolving from positive gains to negative losses through dispersive profiles, upon scanning the RP wavelength across the resonance profile.

**Figure 1:**
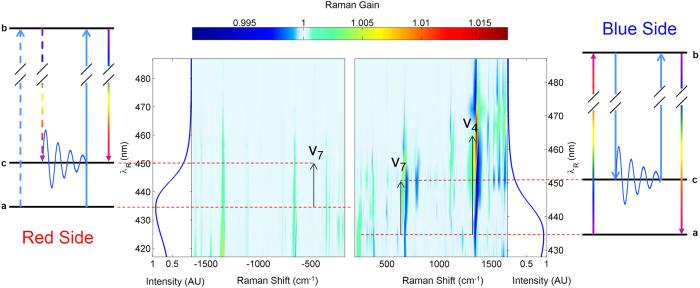
Stimulated Raman spectra of Myoglobin, Central panels: Colormaps of broadband Stimulated Raman spectra, showing the dependence on the Raman pulse wavelength. Signals are always positive in the red side of the spectrum, while in the blue side they show different profiles depending on the resonance condition. Arrows highlights the energies of two prominent vibrational modes (*v*_7_ and *v*_4_), relative to the ground state. Side panels: four-wave mixing energy level diagrams determining Broadband SRS contributes to red (left) and blue (right) sides of the *v*_7_ spectrum.

In Fig. 1 we report SRS spectra for a deoxy Myoglobin sample excited by a RP tuned in the Soret absorption region. The spectra are obtained using a 600 nJ Raman pulse and 50 nJ Probe pulse, both vertically polarised. A description of the experimental setup and data reduction is provided in Methods section, and further detailed in^25^,^26^ and^27^, respectively.

While the red side exhibits a resonance condition with small deviations from the maximum of the Soret band absorption (shown in the side panels of Fig. 1 colormaps)^28^, the maximum Raman gain of the blue side depends on the specific vibrational mode in a much peculiar way and peaks about one vibrational quantum higher in energy than the red side Raman spectrum. In particular, the higher is the frequency of the vibrational level, the lower is the energy of the Raman pulse photon needed to match the resonance. Furthermore, the SRS blue side response changes sign as the full resonant condition is achieved. Indeed, the signal is negative in off-resonant conditions and becomes positive when the Raman pulse is tuned into full resonant conditions, passing through dispersive features. Remarkably, the dependence of the lineshape on the RP wavelength is mode specific as elucidated in Fig. 1. Taking as an example the *v*_4_ Raman band (energy mode ≈1355 cm^−1^), a positive Lorentzian lineshape is observed upon tuning the RP at 465 nm; the transition to a weak negative band occurs by red-shifting or blue-shifting the RP of ≈20 nm relative to the full resonance condition at 465 nm. The *v*_4_ dispersive lineshape arising at RP intermediate wavelengths is characterized by an odd-symmetry: while red-shifting the RP the positive lobe is at higher frequency, a blue-shift of the RP generates a negative lobe at higher frequency. A similar trend occurs for the *v*_7_ mode, taking into account a wavelength shift due to the mechanism elucidated in Fig. 1.

The third order polarization induced in the sample, responsible for the SRG signal, can be evaluated by a perturbative expansion of the density matrix in powers of the electric fields^29^,^30^,^31^; due to the presence a narrowband Raman pulse and a femtosecond Probe pulse one needs to account for several possible field permutations, generating different contributions to the total *P*^(3)^. However, we found that our spectra can be reproduced by a single dominant four-wave mixing contribution for each side of the spectrum, as elucidated by the diagrams in Fig. 1. These diagrams depict the evolution of the density matrix describing the material during the interaction with the electromagnetic fields. On the red side, RP interacts with the medium and promotes first the bra side of the density matrix to the electronic excited state; then a downward interaction with the PP leads the bra in a vibrationally excited (n = 1) electronic ground state, inducing a vibrational coherence, which is then detected through a further interaction with the RP on the ket side of the density matrix and a final free induction decay. Here the convention to calculate only diagrams with the last interaction on the ket side of the density matrix is exploited to halve the computational effort; the remaining diagrams with the last interaction with the bra represent in time domain the complex conjugate of terms in Fig. 1. In the dominant blue side diagram (Fig. 1), the fields only interact with the ket side of the density matrix: the first interaction is with the PP, which promotes the ground state 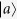 ket to the electronic excited state 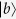. Then the RP induces the vibrational coherence 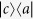, which can be probed after a third interaction with RP and a final free induction decay. As clarified by the diagram, the blue side contribution does not involve a transition starting from a vibrational excited level, therefore the process does not correspond to an Antistokes transition as in Spontaneous Raman spectroscopy^20^.

**Figure 2:**
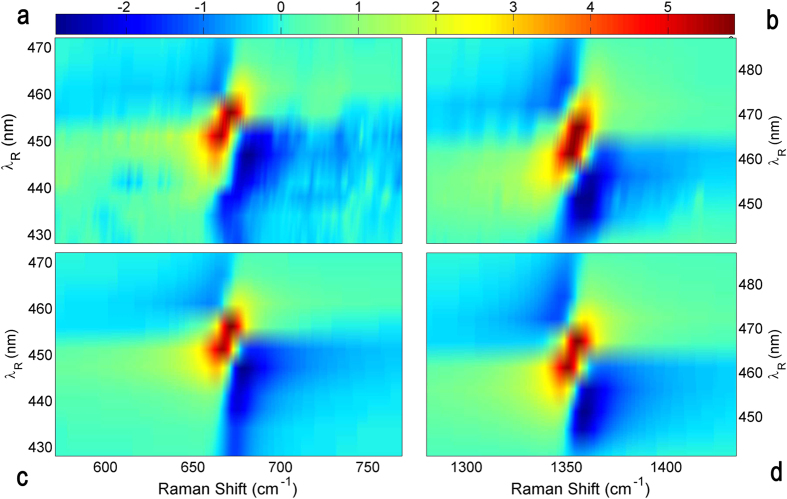
Wavelength dependence of the SRS blue spectra. Experimental broadband SRS data of the blue side broadband Stimulated Raman spectra for the *v*_7_ (**a**) and *v*_4_ (**b**) vibrational modes of Mb. The signal is negative in off-resonant conditions, turns positive by tuning the Raman pulse in full resonant condition, going through dispersive features in between. (**c**,**d**): signal reconstruction obtained from Eq. 1.

Figure 2a,b show a magnification of the blue side SRS spectrum around the *v*_7_ and the *v*_4_ modes of Myoglobin for different Raman pulse wavelengths *λ*_*R*_. Our analysis is reported for these two Myoglobin modes, since they are the most prominent and biochemically relevant (two highly symmetric stretching of the heme ring), but naturally extends to the entire SRS spectrum.

To understand the peculiar behavior of the blue side SRS spectra corresponding to those two modes, we modeled the data by evaluating in the frequency domain the nonlinear response function associated with the two diagrams in Fig. 1.

In Fig. 2c,d we report the calculated *v*_7_ and *v*_4_ SRS bands for the blue side spectra, as a function of the RP wavelength *λ*_*R*_. The model function for the frequency (*ω*) dispersed SRS spectrum is


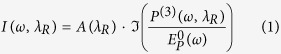


where 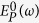 indicates the PP in absence of RP and *A*(*λ*_*R*_) is a positive adjustable parameter which accounts for the effect of the Raman Excitation Profile. The polarizations *P*^(3)^ (*ω*, *λ*_*R*_) for each side of the spectrum are directly obtained from the diagrams in Fig. 1. Writing the Probe and the Raman pulses as 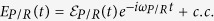, we get


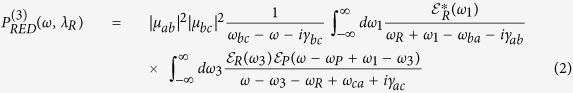


for the red side, and


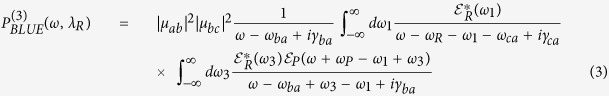


for the blue side. In Eqs. 2 and 3, *μ*_*ij*_ is the dipole transition moment between the *i* and *j* states, *ω*_*ij*_ = *ω*_*i*_ − *ω*_*j*_ is the frequency difference between levels *i* and *j*, and *γ*_*ij*_ is the vibrational dephasing rate of the 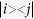 induced coherence. Labels *a*, *b* and *c* indicate respectively the ground state, the excited electronic state and a generic vibrational excited state (on the electronic ground state). A derivation of Eqs. 2 and 3 is reported in section *“Third order nonlinear polarization”* of Supplementary Information. The Raman pulse 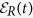 and the Raman probe 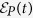 temporal envelopes are modeled as Gaussian profiles:


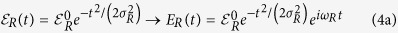



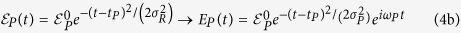


where *t*_*P*_ is the time delay between the Raman pulse and the Raman probe. 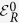, *ω*_*R*_ and *σ*_*R*_ appearing in Eqs. 4a and 4b are calibrated using Cyclohexane SRS spectra and a detailed explanation is provided in Supplementary and in Fig. S2. Thanks to this procedure, only the parameters *A*(*λ*_*R*_), *ω*_*ba*_,*γ*_*ba*_, *μ*_*ba*_, *μ*_*bc*_, *ω*_*ca*_ and *γ*_*ca*_ of the Myoglobin are extrapolated by a global fit through Eqs. 1, 2 and 3.

To simplify the evaluation of Eqs. 2 and 3 and readily interpret the lineshapes of blue and red sides of the spectrum, we consider the simplified case of a monochromatic RP and a continuum (temporal delta function) PP, reducing Eqs. 2 and 3 to


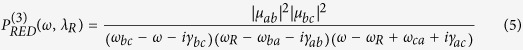


and


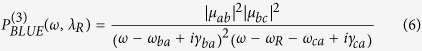


A careful inspection of Eqs. 5 and 6 allows clarifying the effect of the resonance condition on the red and the blue sides. In the former case, the resonance condition arises for *ω*_*R*_ = *ω*_*ba*_, in analogy with SR. In the latter case, the resonance condition depends on the energy of the vibrational mode *ω*_*ca*_ involved in the process, being *ω*_*R*_ = *ω*_*ba*_ − *ω*_*ca*_.

**Figure 3:**
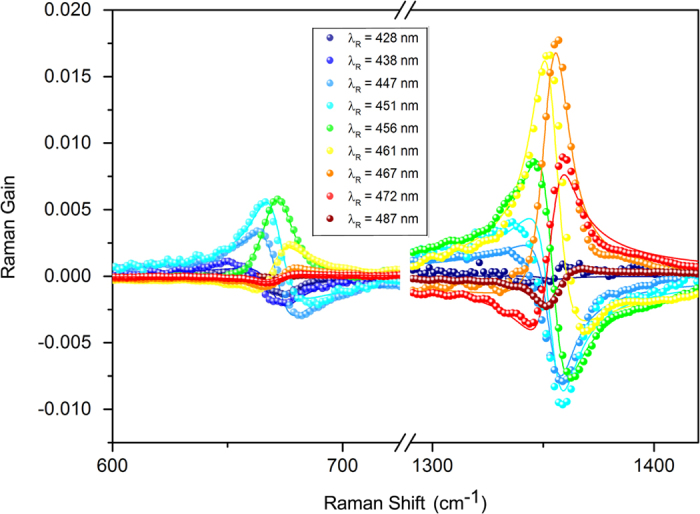
Wavelength dependence of the SRS blue side, Data and Model: Measured SRS lineshapes in the blue side of the Mb *v*_7_ (670 cm^−1^) and *v*_4_ (1355 cm^−1^) Raman modes, represented with dots. In full off-resonance condition (428 and 472 nm for the *v*_7_ mode and 438 or 487 nm for the *v*_4_) the signal is a weak loss, while in full resonance condition (i.e. 456 and 467 nm for *v*_7_ and *v*_4_ respectively) the signal turns into a much more intense gain. The transition between gain and loss proceeds through a “semi-resonant” condition, resulting in dispersive lineshapes. The straight lines represent the model profile obtained by Eq. 1.

**Figure 4:**
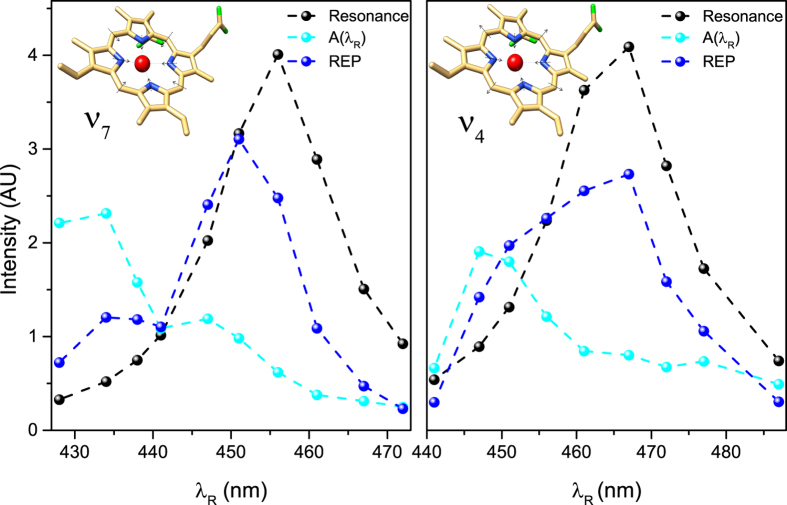
Raman Excitation Profiles for *v*_7_ and *v*_4_ Raman modes. The resonance effects are taken into account in our model by two contributions: a Lorentzian absorption effect (black lines) and the *A*(*λ*_*R*_) coefficient -see Eq. 1- (cyan lines). The resulting REPs are then obtained by the product of these effects and is represented by the blue lines for the *v*_7_ and *v*_4_ Raman modes.

The increase or decrease of photon number in the probe pulse, corresponding to positive or negative Raman bands, is ruled by the sign of the imaginary part of the *P*^(3)^ (*ω*), as shown by Eq. 1. In the red side, the imaginary part’s sign calculated through the diagram on the left of Fig. 1 does not depend on *λ*_*R*_, and it is always positive. Hence the SRG lineshape is a peak for any Raman Pump wavelength. The most interesting case is represented by the blue side: in Eq. 6, as deepened in Supplementary Information, the electronic resonance gives a positive and a negative sign in full off-resonant and full resonant conditions, respectively. This result rationalizes the behavior of the blue side of the spectrum. As it is shown in Fig. 3 the SRS spectra are well reproduced by the model (Eq. 1) for all the Raman pulse wavelengths *λ*_*R*_, using *ω*_*ca*_ = 1354 cm^−1^ and *γ*_*ca*_ = 10 cm^−1^ for the *v*_4_ and *ω*_*ca*_ = 671 cm^−1^ and *γ*_*ca*_ = 10.5 cm^−1^ for the *v*_7_ Raman mode. The parameters *ω*_*ba*_, *γ*_*ba*_ and *A*(*λ*_*R*_) have been adjusted to best fit the experimental data, which enables the possibility to evaluate the blue side Stimulated Raman Excitation Profile (see Fig. 4) for all the Raman active modes, as:


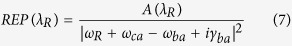


*ω*_*ba*_ and *γ*_*ba*_ model a simple Lorentzian absorption (see Eqs. 5 and 6), and possible deviations from this profile are accounted for by the real positive correction factor *A*(*λ*_*R*_).

**Figure 5:**
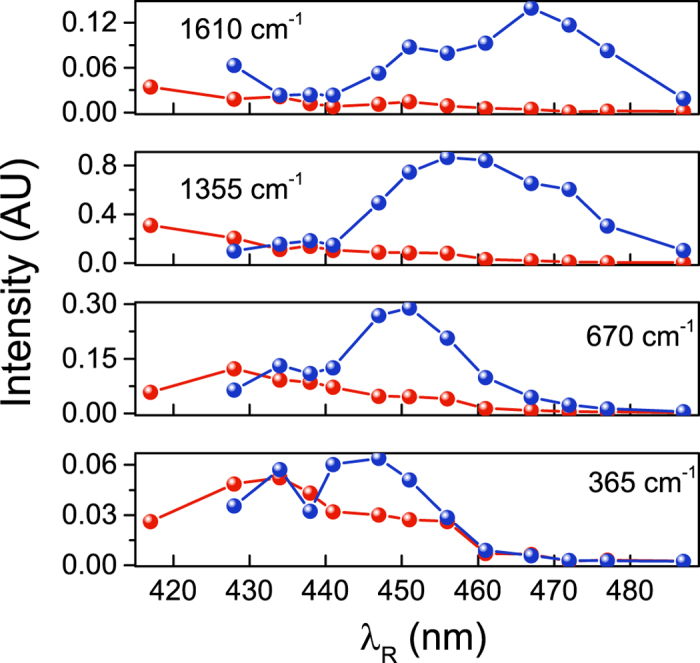
Wavelength intensity SRS spectra dependence: Comparison of spectral intensities in the red side (red lines) and in the blue side (blue lines), for 4 different Raman modes at 365, 670, 1355 and 1610 cm^−1^, evaluated as the integral of the absolute value of the SRS signal of each mode.

Moreover our results allow to understand why, under resonance condition, the blue side of the spectrum shows more intense Raman bands, providing better signal to noise ratio. In the red side, the highest Raman gain intensity is achieved for a RP tuned to the maximum of the electronic absorption. Consequently, while traveling through the sample, RP experiences the maximum depletion due to absorption, and the effective intensity contributing to SRS generation is consequently reduced. Critically, in the blue side, the resonance condition is shifted out of the electronic absorption peak by an amount *ω*_*ca*_. As a consequence, the larger is the energy of the vibrational mode the less is the absorption-induced depletion of the RP intensity in the sample. A higher effective intensity of the RP, contributing to *P*^(3)^ processes, naturally generates more intense SRS signals. The scenario depicted above, is well illustrated in Fig. 5, which reports the experimentally determined SRS band intensities for vibrational modes of different frequency. For each mode the intensity is evaluated as the integral of the absolute value of SRS signal: by tuning the RP, we clearly observe an overall increase of the blue side resonance profile relative to the red side. Furthermore, the wavelength dependence of the blue side spectral intensities displays the already mentioned shift of the resonance condition with the RP wavelength, dependent on the specific vibrational frequency, to be contrasted with a substantial mode independent profile of the red side. A careful inspection of this latter profile, however, reveals an opposite small blueshift. The origin of this behavior is likely to be analogous of the REP which has been analytically evaluated and discussed in ref. 32.

## Conclusions

We reported broadband SRS spectra of Myoglobin, exploring the resonance enhancement across the Soret absorption band. The SRS technique allows obtaining highly resolved vibrational spectra from 200 to 2000 cm^−1^, free from fluorescence background in both the red and the blue side of the spectrum. For the red side, a substantial equivalence between SRS and Spontaneous Raman Stokes side has been verified. In the blue side, dispersive and negative lineshapes have been observed. Positive peaks only arise when the resonance is exactly matched, and this condition critically depends on the frequency of the observed molecular vibration. Importantly, the application of SRS as a probe for femtosecond time-resolved studies (FSRS) requires a judicious account of such complex lineshapes, since further dispersive behaviour can originate in that case from the dynamics of the system. A quantum treatment of the sample through a density matrix formalism allows to identify the contributions of the third order *P*^(3)^ polarization which generate Stimulated Raman bands, reproducing the experimental data.

The proposed approach has been applied to interpret the Myoglobin SRS spectra as an example, but has to be seen as a general procedure to extract Stimulated Raman Excitation Profiles from complex spectral lineshapes. The possibility to selectively enhance different Raman bands, combined with both vibrational and electronic sensitivity, identifies SRS as a formidable tool to investigate Frank-Condon effects and to extract quantitative informations about vibrational mode displacement and vibronic coupling.

The larger cross section under resonance conditions suggests that the blue side Stimulated Raman spectra may be a better candidate to exploit Raman signal, provided that the Raman wavelength lineshape dependence of the spectra is taken into account.

## Methods

### Sample Preparation

Horse heart Myoglobine was purchased from Sigma Aldrich. The commercial protein is in the Fe(III) oxidation state (generally called “ferric form”) which is the form stable in contact with air. The freeze-dried protein was dissolved in pH 7.4 buffer and the obtained solution was purified into a biochemical basket centrifuge.

The final 300 *μ*M Myoglobine solution was prepared anaerobically in a transmission cell under a pure nitrogen atmosphere to prevent the binding between the heme and the atmospheric oxygen, using previously degassed 100 mM pH 7.4 phosfate buffer. A sodium dithionite solution is added to reduce the heme group to the Fe(II) oxidation state (“ferrous form”).

The sample is allowed to flow anaerobically through the transmission cell during the experiment due to a peristaltic pump, so as to guarantee fresh sample at every laser shot (1 KHz). All Raman measurements were performed at room temperaure.

### Experimental setup

A Ti:sapphire laser generates 3.6 mJ, 35 fs pulses at 800 nm and 1 kHz repetition rate. The Raman pulses are synthesized from a two-stage OPA that produces tunable IR-visible pulses, followed by a spectral compression stage based on frequency doubling in a 25 mm Beta Barium Borate (BBO) crystal^33^. Vertically polarized pulses with 10 cm^−1^ bandwidths and 600 nJ intensities are obtained. The femtosecond probe is a vertically polarized white-light continuum (WLC) generated by focusing the laser fundamental into a Sapphire crystal. The Raman features arise on top of the transmitted WLC, which is frequency dispersed by a spectrometer onto a CCD device. A synchronized chopper blocks alternating RP pulses in order to obtain the Raman gain using successive probe pulses. Indicating the *PP* spectrum with and without the presence of the RP as *E*_*P*_ and 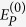, the Raman Gain is obtained as 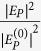.

## Additional Information

**How to cite this article**: Batignani, G. *et al.* Electronic resonances in broadband stimulated Raman spectroscopy. *Sci. Rep.* **6**, 18445; doi: 10.1038/srep18445 (2016).

## Supplementary Material

Supplementary Information
