# Supplementary material for: Electronic resonances in broadband stimulated Raman spectroscopy
Source: Sci Rep. 2016 Jan 5;6:18445. doi: 10.1038/srep18445 (PMC4700463; doi:10.1038/srep18445)
Supplement: Supplementary Information [file srep18445-s1.pdf]

Electronic resonances in broadband stimulated Raman spectroscopy: Supplementary Information

G. Batignani[1,2], E. Pontecorvo[1], G. Giovannetti[1], C. Ferrante[1], G. Fumero[1], T. Scopigno[1,3]

[1]*Dipartimento di Fisica, Universitá di Roma "La Sapienza", I-00185, Roma, Italy*

[2]*Dipartimento di Scienze Fisiche e Chimiche, Universitá degli Studi dell'Aquila , I-67100, L'Aquila, Italy and*

[3] *Center for Life Nano Science @Sapienza, Istituto Italiano di Tecnologia, 295 Viale Regina Elena, I-00161, Roma, Italy*

(Dated: September 23, 2015)

Third order nonlinear polarization

The complete third order response of the system is given by

$$P^{(3)}(t) = \int_0^\infty d\tau_3 \int_0^\infty d\tau_2 \int_0^\infty d\tau_1 E(t-\tau_3)E(t-\tau_2-\tau_3)E(t-\tau_1-\tau_2-\tau_3)S^{(3)}(\tau_1,\tau_2,\tau_3) \quad \text{(S1)}$$

where $S^{(3)}(\tau_1,\tau_2,\tau_3)$ is

$$S^{(3)}(\tau_1,\tau_2,\tau_3) = \left(-\frac{i}{\hbar}\right)^3 Tr\left(\mu(\tau_1+\tau_2+\tau_3)\left[\mu(\tau_1+\tau_2),[\mu(\tau_1),[\mu(0),\rho(-\infty)]]\right]\right) \quad \text{(S2)}$$

and $E(t) = \sum_i \mathcal{E}_i(t)e^{-i\omega_i t} + c.c.$.

By the use of energy-level diagrams, two contributions can be isolated to reproduce the stimulated Raman signal in the red and blue side of the Raman pulse (see. Fig 1).

$$P^{(3)}_{RED}(t) = \left(-\frac{i}{\hbar}\right)^3 |\mu_{ab}|^2|\mu_{bc}|^2 \int_0^\infty d\tau_3 \int_0^\infty d\tau_2 \int_0^\infty d\tau_1 \mathcal{E}^*_R(t-\tau_1-\tau_2-\tau_3)\mathcal{E}_P(t-\tau_2-\tau_3)\mathcal{E}_R(t-\tau_3)$$
$$e^{i\omega_R(t-\tau_1-\tau_2-\tau_3)}e^{-i\omega_P(t-\tau_2-\tau_3)}e^{-i\omega_R(t-\tau_3)}e^{-i\tilde{\omega}_{ab}\tau_1}e^{-i\tilde{\omega}_{ac}\tau_2}e^{-i\tilde{\omega}_{bc}\tau_3} \quad \text{(S3)}$$

$$P^{(3)}_{BLUE}(t) = \left(-\frac{i}{\hbar}\right)^3 |\mu_{ab}|^2|\mu_{bc}|^2 \int_0^\infty d\tau_3 \int_0^\infty d\tau_2 \int_0^\infty d\tau_1 \mathcal{E}_P(t-\tau_1-\tau_2-\tau_3)\mathcal{E}^*_R(t-\tau_2-\tau_3)\mathcal{E}_R(t-\tau_3)$$
$$e^{-i\omega_P(t-\tau_1-\tau_2-\tau_3)}e^{i\omega_R(t-\tau_2-\tau_3)}e^{-i\omega_R(t-\tau_3)}e^{-i\tilde{\omega}_{ba}\tau_1}e^{-i\tilde{\omega}_{ca}\tau_2}e^{-i\tilde{\omega}_{cb}\tau_3} \quad \text{(S4)}$$

where $\tilde{\omega}_{ij} = \omega_i - \omega_j - i\gamma_{ij}$.

The frequency dispersed signal can simply be calculated by the Fourier transform $P^{(3)}(\omega) = \int_{-\infty}^{\infty} P^{(3)}(t)e^{i\omega t}dt$.

To reduce the computational effort to calculate S3 and S4 it is useful to write the pulse field in terms of their Fourier transform

$$P^{(3)}_{RED}(\omega) = \left(-\frac{i}{\hbar}\right)^3 |\mu_{ab}|^2|\mu_{bc}|^2 \int_{-\infty}^\infty dt\, e^{i\omega t} \int_0^\infty d\tau_3 \int_0^\infty d\tau_2 \int_0^\infty d\tau_1 \int_{-\infty}^\infty d\omega_1 \int_{-\infty}^\infty d\omega_2 \int_{-\infty}^\infty d\omega_3$$
$$\mathcal{E}^*_R(\omega_1)e^{i(\omega_R+\omega_1)(t-\tau_1-\tau_2-\tau_3)}\mathcal{E}_P(\omega_2)e^{-i(\omega_P+\omega_2)(t-\tau_2-\tau_3)}\mathcal{E}_R(\omega_3)e^{-i(\omega_R+\omega_3)(t-\tau_3)}e^{-i\tilde{\omega}_{ab}\tau_1}e^{-i\tilde{\omega}_{ac}\tau_2}e^{-i\tilde{\omega}_{bc}\tau_3} \quad \text{(S5)}$$

In this way all the temporal integral may be solved analytically.

$$P^{(3)}_{RED}(\omega) = |\mu_{ab}|^2|\mu_{bc}|^2 \frac{1}{\tilde{\omega}_{bc}-\omega} \int_{-\infty}^\infty d\omega_1 \frac{\mathcal{E}^*_R(\omega_1)}{\omega_R+\omega_1+\tilde{\omega}_{ab}} \int_{-\infty}^\infty d\omega_3 \frac{\mathcal{E}_R(\omega_3)\mathcal{E}_P(\omega-\omega_P+\omega_1-\omega_3)}{\omega-\omega_3-\omega_R-\tilde{\omega}_{ac}} \quad \text{(S6)}$$

	Off-Resonance	Resonance
Red Side	$(\omega_{bc}-\omega)\,(\omega_R-\omega_{ba})$	$(-i\gamma_{bc})\,(-i\gamma_{ab})$
Blue Side	$(\omega-\omega_{ba})^2$	$(i\gamma_{ba})^2$

TABLE I: Denominator of electronic factors in Eqs. S10 and S11, for the red and the blue side under resonance and off-resonance condition.

where we used the conservation of energy $\int_{-\infty}^{\infty} e^{i(\omega-\omega_P+\omega_1-\omega_3-\omega_2)t}dt = \delta(\omega-\omega_P+\omega_1-\omega_3-\omega_2)$ to simplify the ω_2 integral.

$$P^{(3)}_{BLUE}(\omega) = |\mu_{ab}|^2|\mu_{bc}|^2\frac{1}{\omega-\tilde{\omega}_{ba}}\int_{-\infty}^{\infty}d\omega_1\frac{\mathcal{E}_R(\omega_1)}{\omega-\omega_R-\omega_1-\tilde{\omega}_{ca}}\int_{-\infty}^{\infty}d\omega_3\frac{\mathcal{E}^*_R(\omega_3)\mathcal{E}_P(\omega+\omega_P-\omega_1+\omega_3)}{\omega-\tilde{\omega}_{ba}+\omega_3-\omega_1} \quad \text{(S7)}$$

By considering monochromatic RP and continuous PP, Eqs. S6 and S7 may be recast as

$$P^{(3)}_{RED}(\omega) = \frac{|\mu_{ab}|^2|\mu_{bc}|^2}{(\omega_{bc}-\omega-i\gamma_{bc})\,(\omega_R-\omega_{ba}-i\gamma_{ab})\,(\omega-\omega_R+\omega_{ca}+i\gamma_{ac})} \quad \text{(S8)}$$

and

$$P^{(3)}_{BLUE}(\omega,\lambda_R) = \frac{|\mu_{ab}|^2|\mu_{bc}|^2}{(\omega-\omega_{ba}+i\gamma_{ba})^2\,(\omega-\omega_R-\omega_{ca}+i\gamma_{ca})} \quad \text{(S9)}$$

The electronic resonance enhancement is contained in

$$\frac{1}{(\omega_{bc}-\omega-i\gamma_{bc})\,(\omega_R-\omega_{ba}-i\gamma_{ab})} \quad \text{(S10)}$$

for the red side, and

$$\frac{1}{(\omega-\omega_{ba}+i\gamma_{ba})^2} \quad \text{(S11)}$$

for the blue side. The denominator of the former contribution can be approximated as shown in table I. Remarkably, while in the red side of the spectrum the electronic resonance factor gives always the same sign to the total $P^{(3)}$, in the blue side it is positive under resonance condition and negative under the off-resonance one. The sign on the off resonance red side can be easily evaluated by the substitution $(\omega_{bc}-\omega)\approx(\omega_{bc}-(\omega_R-\omega_{ca}))=(\omega_{bc}-\omega_R+\omega_{ca})=(\omega_{ba}-\omega_R)$, from which it follows $(\omega_{bc}-\omega)\,(\omega_R-\omega_{ba})\approx-(\omega_R-\omega_{ba})^2$.

The Stimulated Raman Excitation Profile $REP(\lambda_R)=\frac{A(\lambda_R)}{|\omega_R+\omega_{ca}-\omega_{ba}+i\gamma_{ba}|^2}$ in Eq. 7 is obtained combining Eqs. 1 and S11.

Fit Procedure and Data Treatment

To isolate the Raman contributions in Myoglobin SRS spectra a background signal has to be removed. Transient absorption of the Raman pulse and additional nonlinear contributions from both the solute and the solvent generate a broad baseline signal, which can be removed adding a low (second or third) order polynomial to the fit procedure presented previously (Eq. 1). In Fig. S1 we report the polynomial background signal obtained in the fit procedure for the high frequency region around the ν_4 mode, where the filled area represents the SRS isolated contributions.

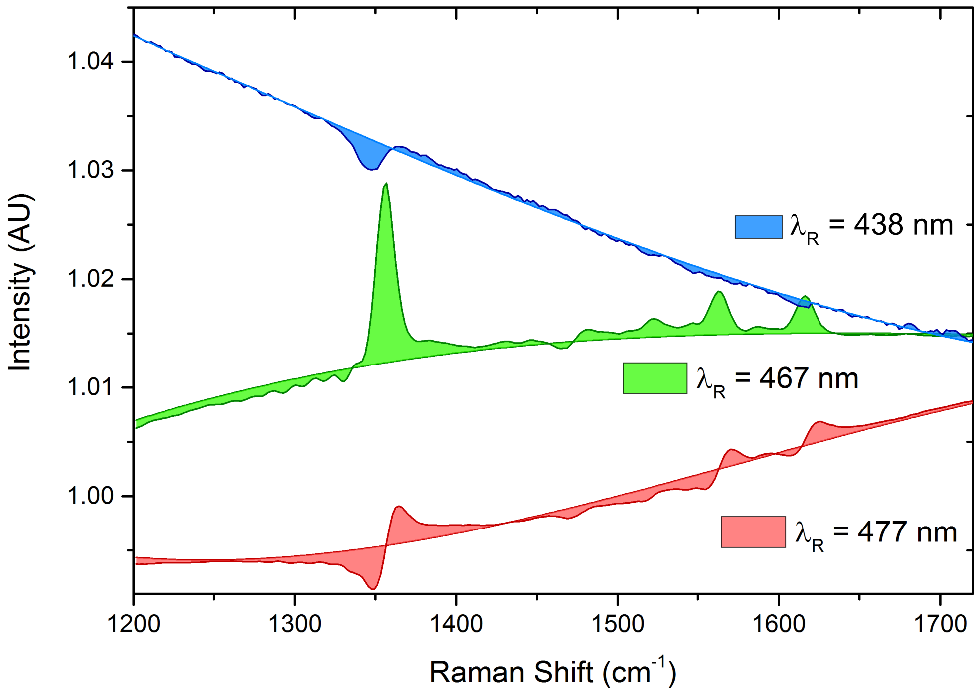

FIG. S1: **Baseline Removal example**: Polynomial background signal obtained in the fit procedure for the high frequency region around the ν_4 mode, where the filled area represent the SRS isolated contributions.

Broadband SRS spectra of Cyclohexane is acquired and used for a calibration of the Raman pulse and Raman probe parameters (see Eq. 4). The precise narrow-band Raman pulse wavelength (ω_R) and its intensity $\mathcal{E}_R^0$ are measured by fitting the Cyclohexane Raman modes position and intensity, while the Raman pulse time duration (σ_R) and the delay t_P are obtained by the same fit procedure on the more resolved 800 cm^{-1} Cyclohexane Raman mode, using Eq. S6, as shown in Fig. S2.

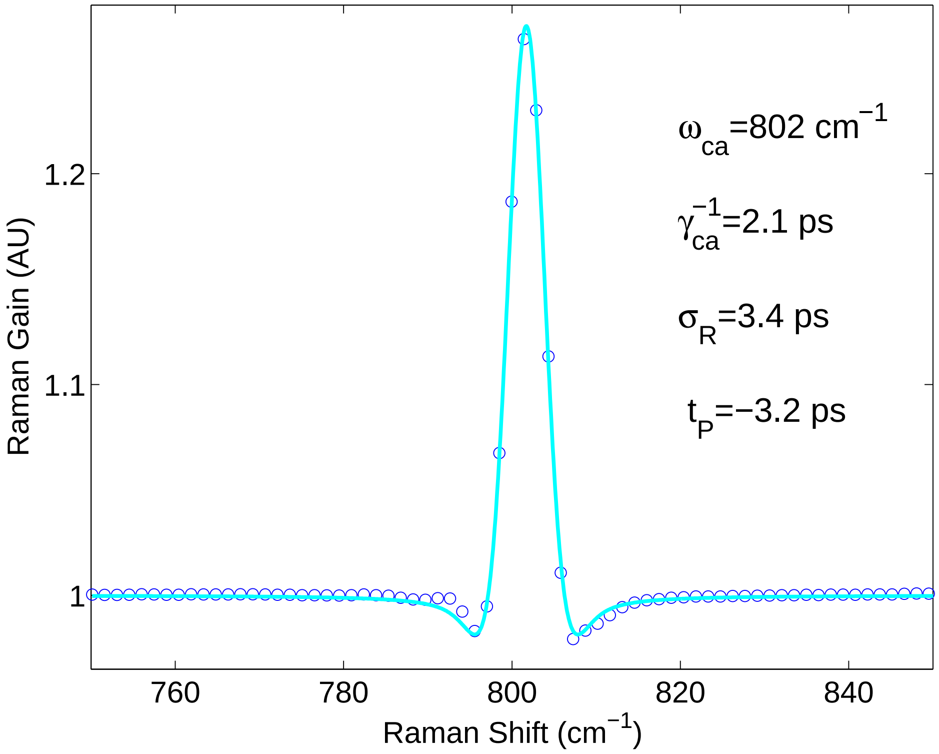

FIG. S2: **Cyclohexane Calibration**: The Cyclohexane spectrum is fitted to calibrate the Raman pulse duration σ_R and its time delay t_P with the probe pulse. In the figure we report these parameters together with the fitted peak position ω_{ca} and the bandwidth γ_{ca} of the reported mode. The small negative side wings indicate that the PP is preceding the RP, ensuring the best condition in terms of spectral resolution and maximum of Raman Gain[1].

---

1. Yoon, S. *et al.* Dependence of line shapes in femtosecond broadband stimulated raman spectroscopy on pump-probe time delay. *J. Chem. Phys* **122**, 024505 (2005).
